# New Treatment Strategies for Alcohol-Induced Heart Damage

**DOI:** 10.3390/ijms17101651

**Published:** 2016-09-29

**Authors:** Joaquim Fernández-Solà, Ana Planavila Porta

**Affiliations:** 1 Alcohol Unit, Department of Internal Medicine, Hospital Clinic, University of Barcelona, Villarroel 170, 08036 Barcelona, Spain; 2 Departament of Biochemistry and Molecular Biomedicine, Faculty of Biology, Avda Diagonal 643, Universitat de Barcelona, 08028 Barcelona, Spain; aplanavila@ub.edu

**Keywords:** alcoholic cardiomyopathy, apoptosis, fibrosis, hypertrophy, regeneration, cardiomyokines, myostatin, IGF-1, FGF21, miRNA

## Abstract

High-dose alcohol misuse induces multiple noxious cardiac effects, including myocyte hypertrophy and necrosis, interstitial fibrosis, decreased ventricular contraction and ventricle enlargement. These effects produce diastolic and systolic ventricular dysfunction leading to congestive heart failure, arrhythmias and an increased death rate. There are multiple, dose-dependent, synchronic and synergistic mechanisms of alcohol-induced cardiac damage. Ethanol alters membrane permeability and composition, interferes with receptors and intracellular transients, induces oxidative, metabolic and energy damage, decreases protein synthesis, excitation-contraction coupling and increases cell apoptosis. In addition, ethanol decreases myocyte protective and repair mechanisms and their regeneration. Although there are diverse different strategies to directly target alcohol-induced heart damage, they are partially effective, and can only be used as support medication in a multidisciplinary approach. Alcohol abstinence is the preferred goal, but control drinking is useful in alcohol-addicted subjects not able to abstain. Correction of nutrition, ionic and vitamin deficiencies and control of alcohol-related systemic organ damage are compulsory. Recently, several growth factors (myostatin, IGF-1, leptin, ghrelin, miRNA, and ROCK inhibitors) and new cardiomyokines such as FGF21 have been described to regulate cardiac plasticity and decrease cardiac damage, improving cardiac repair mechanisms, and they are promising agents in this field. New potential therapeutic targets aim to control oxidative damage, myocyte hypertrophy, interstitial fibrosis and persistent apoptosis In addition, stem-cell therapy may improve myocyte regeneration. However, these strategies are not yet approved for clinical use.

## 1. Introduction

Alcohol (Ethanol) consumption is one of the major factors inducing cardiac and vascular diseases worldwide, mainly when it is consumed at high-dose in binging, unhealthy use high doses or in unhealthy binge episodes [1,2,3]. At present, alcohol misuse is a major public health concern due to the increase in cardiac morbidity and mortality, in addition to damage to the central nervous system (CNS) and the liver [4]. According to the World Mortality report 2013, cardiovascular diseases are still the main global cause of death, being responsible for one third of total mortality with 3.3 million deaths in 2012 [5]. Therefore, alcohol is considered as one of the six major contributors to cardiovascular damage [4,6]. One study reported that the prevalence of alcohol cardiomyopathy in long-standing alcohol misuse was 13% compared to a matched cohort of non-alcoholics [7]. Although the possible mechanisms of alcohol-induced cardiovascular damage are not fully understood, studies have proposed direct and nutrient-based mechanisms [6,7,8,9]. Major therapeutic approaches in this field involve the avoidance or control of alcohol consumption [10]. However, this is not always possible since a large quantity of alcohol may be consumed to prevent the withdrawal symptoms in addicts. In addition, this strategy does not reverse the permanent damage to the heart produced by unhealthy alcohol consumption [11].

As a plastic organ, the heart is able to partially control and adapt to a damaging toxic agent such as moderate ethanol consumption [3,12]. However, when this aggression is persistent and at high-doses over years, it may overcome the protective mechanisms of the heart of the individual, leading to progressive cardiac damage and ensuing functional dysfunction. Recently, different approaches have been proposed to reduce alcohol-related heart damage [10,11,12,13,14,15]. Therefore, a multi-disciplinary therapeutic approach is needed to increase the protective mechanisms of the heart and thereby improve public health. The aim of this review is to summarize the new multidisciplinary therapeutic strategies under development to decrease or reverse alcohol-induced cardiac damage.

## 2. Mechanism of Alcohol-Induced Cardiac Damage

Ethanol is a highly reactive biologically small-size molecule that easily diffuses through the biological membranes as well as the intracellular compartments, being able to achieve and target all intracellular organelles [8,9]. It interacts with membrane phospholipids, ion channels and receptors, modifying their structure and function, altering intracellular transients as well as cell energy and oxidative status. It is a potent enzymatic inductor and has active metabolites (acetaldehyde-acetate, fatty-acid ethyl esthers) [8,16]. Table 1 summarizes the main pathogenic mechanisms of alcohol-induced heart damage.

In the cardiac cell, alcohol is a sensitizing agent that interferes with cell structures including intracellular membranes, channels, receptors and DNA. In addition, ethanol alters protein synthesis and turnover involving the structural cardiac proteins (actin, myosin, troponin, and titin) and induces alcohol–protein interaction generating adducts and reactive immune complexes producing additional inflammatory damage [1,36]. This myocyte damage by ethanol deregulates cardiac energy production, causing a decrease in the excitation-coupling mechanism that disturbs cardiac contractility. Acute binge and chronic lifetime cumulated cardiac damage may have additive effects [1]. Therefore, the potential damage that ethanol may inflict on cardiac myocytes is multi-factorial, intense and persistent [9].

Table 2 summarizes the diverse harmful cardiovascular effects of high-dose alcohol. In general, alcohol-induced heart damage may involve a myriad of seemingly independently acting molecular signals that may act synchronically and also synergistically. Age, genetic polymorphisms, gender, race and behavioral factors can modify the personal susceptibility to this ethanol-induced cardiac damage [9,16]. Although they are not the main pathologic factors in alcoholic cardiomyopathy (ACM), alcohol-induced vitamins (thiamine, pyridoxine, and cobalamin), cofactors (folate), protein or caloric malnutrition, may increase the severity of heart damage [2,9]. Alcohol consumption can also increase the adverse effects of other cardiac damaging factors causes of heart damage, mainly tobacco and cocaine [19,37]. Therefore, evaluation of alcohol-induced heart damage should be performed in a multidisciplinary and personal basis

## 3. Strategies to Decrease Factors Inducing Heart Damage

Although many strategies have been suggested to decrease heart damage in general [38,39,40,41], they are only partially effective in cases of alcohol-related heart damage. Thus, they can only be considered as complementary treatments. The following paragraphs describe important approaches currently being used to suppress alcohol-induced cardiac disorders.

### 3.1. Control of Alcohol Consumption

Alcohol-induced cardiac damage is directly associated with binge drinking and chronic non-binge drinking in which the lifetime cumulated dose of ethanol is a relevant factor [1,2,3]. Control strategies may involve both drinking modes [1,13,42]. Development of addiction and withdrawal symptoms further complicate treatment approaches [10,11]. Binge drinking, defined as the consumption of more than five alcohol units per occasion, induces an acute decrease of myocyte contractility and arrhythmia and may cause sudden death [43]. In subjects with previously defined heart disease, binge drinking may be a serious issue and should be avoided [1,9]. Success in attempting to abstain from alcohol is only achieved in 40%–50% of long-term alcohol consumers probably due to difficulties in the management of alcohol withdrawal. The latter is a relevant issue to be addressed in order to avoid or decrease persistent alcohol-induced heart damage [11]. Recently, the efficacy of the abstinence approach was evaluated in patients with alcoholic cardiomyopathy [42]. This study showed that: (1) subjects who completely abstained from alcohol showed improvement in cardiovascular function without significant withdrawal symptoms; and (2) those subjects who could not abstain, but were able to significantly decrease their alcohol consumption in a controlled manner (20–60 g/day) also presented major improvement in cardiac function [42]. This suggests that ethanol abstention is the preferred goal in subjects with ACM, although controlled drinking is still a useful strategy in subjects not able to abstain. Any decrease of the quantity of alcohol consumed may be useful to avoid progressive alcohol-induced heart damage.

### 3.2. Comorbid Factors

In addition to alcohol, control of tobacco, cocaine and other unhealthy drug consumption toxic substances is necessary to manage alcohol addiction in a multi-toxic consumption pattern [44,45]. This approach may require a specialized multidisciplinary strategy with personalized cognitive-behavioral psychotherapy and the use of pharmacological support for alcohol and drug addiction [13].

### 3.3. Therapy against Alcohol-Induced Non-Cardiac Systemic Damage

Alcohol is a systemic toxic substance that, in addition to heart damage, induces multiple simultaneous organ-damage, mainly in the liver, brain, muscle, lung, and kidney, and also disturbs the nutritional status [9]. Subclinical systemic organ damage has an independent influence on cardiac risk and may amplify the estimated risk damage [46]. In fact, as previously reported in the literature [47], it is pivotal to consider systemic organ impairment when approaching patients with heart failure, mostly when there is a mutual noxae such as alcohol [46]. Subclinical organ damage is a predictor of cardiovascular death ant its evaluation may improve risk prediction [48]. Similarly, cardiovascular disorders may also themselves induce systemic organ damage involving lung, liver, kidney, and brain dysfunction [49].

The liver is the most important organ affected by ethanol toxicity and is clearly interrelated with heart damage because of some common pathogenic injury mechanisms such as oxidative and inflammatory damage as well as the induction of apoptosis and fibrosis [50]. Uncompensated liver cirrhosis contributes to alterations in cardiac function and should be controlled [51].

After the liver, the brain is the organ most frequently involved in systemic alcohol toxicity causing a wide spectrum of structural and functional changes in addition to alcohol addiction [52]. This produces an important health-related systemic impact [53]. Some alcohol-induced brain diseases such as intracranial hemorrhage produce myocardial damage and cardiac arrhythmia, probably through increased catecholamine production (sympathetic storm) that may also increase sudden death in alcoholism [54]. Due to the known brain-heart health connection, control of the visceral organ dysfunction that occurs as a result of neurological stimuli (neurological heart disease) is also relevant to stabilize alcohol-induced heart damage [55].

The lung is also affected by chronic alcohol injury, increasing the risk of pneumonia, sepsis and acute respiratory distress syndrome (ARDS) [56]. The presence of alcohol-induced lung damage increases oxidative stress and interferes with heart damage, with a raised incidence of coronary heart disease [57].

Skeletal muscle is anatomically and functionally similar to the heart, and there is a clear relationship between cardiac and skeletal damage induced by ethanol [1,28]. In end-stage heart failure, muscle wasting and sarcopenia are relevant factors influencing the quality of life [58]. Therefore, improvement of muscle thophism and strength contributes to improving heart function in chronic alcoholics.

The kidney may also be damaged by high-dose ethanol in experimental [59] and clinical [60] settings. Since kidney dysfunction can cause cardiac overload and increased oxidative stress [61], it is important to control kidney damage in subjects with alcohol-induced heart damage.

The presence of sustained systemic inflammatory response also increases alcohol-induced heart damage [22,27,62], and must be controlled to avoid increased heart-damage.

Caloric and protein malnutrition as well as vitamin deficiencies (i.e., cardiac beriberi) are frequent in chronic alcoholics [51,63,64] and contribute to an increase in alcohol-induced structural and functional cardiac changes [1]. Similarly, ionic disruption including hypo- and hyperkaliemia, hypocalcemia, hypomagnesemia and hyperphosphoremia worsens cardiac contractility and excitability [1,64]. Therefore any additional nutrition, vitamin or ion disturbance should be corrected to stabilize alcohol-mediated heart damage [1,7,9].

Thus, inclusion of systemic organ damage markers in the assessment of alcohol-induced heart damage and control of non-cardiac systemic damage may contribute to improve alcohol-induced heart damage.

### 3.4. Therapeutic Approaches against Myocyte Hypertrophy and Cell Loss

Cardiac hypertrophy increases cardiomyocyte size and myocardial mass in response to physiological or pathological events that also induce remodeling [65]. Myocyte hypertrophy is a key factor in the transition from a normal to a pathologic heart in alcohol-induced and other causes of heart damage [28,66]. However, cardiac hypertrophy may also be an adaptive mechanism to stressful conditions of the heart, but prolonged hypertrophy may lead to cardiac dysfunction and heart failure which represents the primary cause of human morbidity and mortality [65,67]. According to the Frank–Starling law, mechanical overload to the ventricle is compensated with chamber enlargement [66,68]. This process is also accompanied by concentric hypertrophy that may progress to outflow obstruction [67]. In the case of alcohol, ventricular enlargement is eccentric, with preservation of septum thickness, thus avoiding blood-flow obstruction [3,29].

Factors associated with pathological myocyte hypertrophy and abnormal remodeling are cytokine-mediated inflammation, disruption of intracellular transduction signals, angiogenesis and interactions with other cells mediated by the autonomic system [66,69]. In addition, epigenetic factors may also modulate this process [67]. Myocyte loss by necrosis or apoptosis is a relevant mechanism in cardiac dysfunction and is present in major heart diseases [70]. High-dose alcohol consumption has clearly shown to increase cardiac apoptosis pathways in animal [23] and human studies [24]. Thus, pro-apoptotic mechanisms are activated in alcoholic patients without heart damage. Chronic alcoholic subjects with structural heart damage showed higher apoptotic indexes in deoxyribonucleotidyl transferase-mediated dUTP-biotin nick end-labeling, Bax, and Bcl-2 assays as compared with control subjects [23,24]. Since the control of myocyte apoptosis is a key factor in alcohol-induced heart damage [9], the most relevant strategies to control cardiac hypertrophy and myocyte loss include the following.

#### 3.4.1. Myostatin (Mstn)

Mstn regulation has been proposed to control excessive myocyte hypertrophy. Mstn is the growth and differentiation factor 8 (GDF-8), a member of the transforming growth factor-β superfamily of growth factors which acts as the negative regulator of skeletal muscle and cardiac growth [71]. Mstn activity protects cardiac cells from apoptosis [72]. However, Mstn has other cardiac effects, increasing hypertrophy and also inhibiting myocyte proliferation. In fact, Mstn represses AMPK activation of TAK1, restricting hypertrophy and regulating cardiac metabolism interacting with key metabolic enzymes [73]. Adult- versus embryonic-specific inactivation of Mstn has a different effect. In adults, Mstn inactivation induces excessive expression in non-cardiomyocytes in the heart and rescues hypertrophy in aging mice, confirming the role of Mstn in the regulation of cardiac hypertrophy [74]. Therefore, Mstn, as well as its analog GDF11, have anti-hypertrophic and cardio-protective mechanisms, necessary to maintain aerobic energy metabolism in adult cardiomyocytes [75].

Earlier experimental animal studies have shown a significant increase in cardiac Mstn expression in chronic high-dose alcohol exposure when compared to non-exposed controls, confirming a clear over-expression of Mstn with alcohol consumption [67,76]. This is further confirmed by the observation that, in subjects with alcohol-induced cardiomyopathy, there a significant increase in cardiac Mstn expression in comparison to healthy controls [31]. This Mstn up-regulation has been observed either in alcoholic, hypertensive, valves, ischemic or idiopathic cardiomyopathy (CMP), being independent of the etiologic origin of the CMP [31,77].

To explain the relationship between Mstn and cardiac hypertrophy we should consider that Mstn represses AMPK and interacts with key metabolic proteins and enzymes [78]. Therefore, Mstn up-regulation may be a regulatory mechanism activated to avoid excessive hypertrophy and pathological cardiac remodeling in ethanol-induced cardiac damage [73]. 

#### 3.4.2. Adrenergic Receptors (AR)

Cardiac hypertrophy is regulated by multiple factors with a clear influence of α1-AR. Indeed, the signaling events of this receptor contribute to the definition of molecular and cellular cardiac features [65,79]. New hypotheses have emerged concerning the functional role of α1-AR receptors in the heart. Regulation of these receptors may modify cardiac hypertrophy [65]. Thus, endogenous BNP attenuates cardiomyocyte hypertrophy induced by Ang II via the p38 MAPK/Smad signaling pathway [80,81]. Overstimulation of the Renin-Angiotensin System (RAS) has been implicated in a chain of events that contribute to the pathogenesis of cardiovascular disease and cardiac remodeling. Novel pathways within the RAS and new therapeutic approaches that target this system are required to further reduce Ang II formation, and thereby provide patients with additional benefits from a more complete blockade of the RAS [82]. In addition, leptin antagonist therapy may attenuate angiotensin II-induced LV hypertrophy and has been used in local application [83]. β-3 AR also exerts antioxidant protective effects, improving cardiac hypertrophy and remodeling in response to neuro-hormonal stimulation [84].

#### 3.4.3. Oxidative/Nitrative Stress

Oxidative/Nitrative stress, a key etiological factor in the development of alcohol-induced cardiac toxicity [20], is related to mitochondrial function, myocyte hypertrophy, autophagy and apoptosis [79,85]. Reducing oxidative stress may also contribute to decreasing myocyte hypertrophy as described with the use of the non-peptide angiotensin-(1-7) analogous AVE 0991. In fact, AVE 0991 significantly down-regulates the mean diameter of myocytes, inhibits NOX2 and NOX4 expression and attenuates gene expression of the hypertrophic markers. AVE 0991 treatment could attenuate cardiac hypertrophy and improve heart function, which may be due to reduce oxidative stress [86]. Therefore, treatments addressed at reducing heart oxidative stress may be a useful complementary approach to consider in order to reduce unhealthy cardiac hypertrophy.

Epigallocatechin gallate (EGCG) is a polyphenol derived from green tea. It has a wide range of biological activities, including antioxidant and anti-apoptotic activities though the PI3K/Akt signaling pathway. EGCG post-conditioning inhibits myocardial apoptosis and restores the autophagic flux, suggesting that it may be useful as an anti-apoptotic cardioprotective agent [87].

#### 3.4.4. Myocardium RhoA/ROCK Pathway

The Rho-associated coiled-coil containing kinases (ROCKs) are members of the serine/threonine protein kinase family, which mediates the downstream effects of the small GTP-binding protein RhoA [88]. These pathways contribute to cardiac remodeling induced by persistent hypertrophic stress, leading to heart dysfunction and failure. As evidenced in experimental studies, the use of RhoA/ROCK inhibitors is a potential cardiac target to avoid hypertrophy [89]. Stretch-induced activation of RhoA differentially regulates angiotensinogen gene expression by modulating p38 and JNK activation [90]. RhoA/ROCK also regulates an apoptotic response depending on the cell type and the apoptotic stimulus. Acute RhoA/ROCK activation inhibits apoptosis through the FAK/PI3K/Akt survival pathway. Conversely, more sustained activation of Rho/ROCK (48–72 h) induces apoptosis through activation of p53/Bax-mediated mitochondrial death pathway. The anti-apoptotic effects of ROCK1 deletion were found to be associated with enhanced ERK/MAPK and/or Akt activation. This suggests a role for ROCK1 in modulating the activity of these survival pathways under pathological conditions that may contribute to cardiac remodeling [89].

Studies have shown that Azaindole-1, a novel RhoA/ROCK ATP-competitive inhibitor, has blood pressure lowering effects [91]. SLx-2119 and Fasudil competitively bind to the ROCK ATP pocket [92]. These inhibitors block the generation of inflammatory cytokines, such as interleukin-6 and tumor necrosis factor-alpha and induce vasorelaxation. Fasudil is the only ROCK inhibitor approved for human use for the prevention and treatment of cerebral vasospasm. Given the safety and effectiveness and extensive preclinical data described in experimental model systems, small clinical trials have been carried out and have demonstrated some of the benefits of fasudil in cardiac hypertrophy [88,92]. The future development and application of isoform-specific ROCK inhibitors in knockout animal models as well as human clinical trials are expected [89].

#### 3.4.5. Sirtuins

Sirtuins are the Sir2A family of class III histone deacetylases [93] that have recently been involved in a wide range of physiological and pathological processes, including aging, energy regulation, as well as cardiac hypertrophy, apoptosis and inflammation. Specifically, Sirt7 is known to regulate heart apoptosis and stress responses [94]. Sirt3 protects cardiomyocytes from oxidative stress and suppresses cardiac hypertrophy [94]. Sirt1, Sirt3 and Sirt6 have also demonstrated to attenuate cardiac hypertrophy, and protect cardiomyocytes from aging and oxidative stress [94,95]. Resveratrol is a natural Sirt-1-specific activator that also exerts cardio-protective effects that regulate redox signaling during oxidative stress in cardiovascular disease. Resveratrol-regulated autophagy may play a role in degrading damaged organelles, thus improving cardiac function [96]. The administration of resveratrol prevents the alteration in SIRT-1 in type-2 diabetes mellitus and SIRT-1, 2, 3 and SIRT-5 in the type 1 diabetes mellitus rat heart [97].

#### 3.4.6. Caspase Inhibition

Since caspases are pro-apoptotic mitochondrial inductors, inhibition of caspase 3 activity has been proposed as an anti-apoptotic treatment. Hesperetin a flavonone glycoside has been proposed to attenuate mitochondria-dependent cardiac apoptosis [98].

#### 3.4.7. Suppressor of IKKε (SIKE)

Another potential mechanism to treat cardiac hypertrophy, remodeling and heart failure is inhibition of the TBK1/AKT pathway. SIKE, a suppressor of IKKε, produces negative regulation of the interferon pathway and regulates cardiac remodeling [99]. Sike-deficient mice develop cardiac hypertrophy, whereas sike-overexpressing transgenic (Sike-TG) mice are protected from hypertrophic stimuli. Due to its inhibitory regulation of the TBK1/AKT axis, SIKE has been proposed as a negative regulator of cardiac remodeling in multiple animal species, and may represent a therapeutic target for the treatment of cardiac hypertrophy and heart failure [99].

#### 3.4.8. Micro RNA (miRNAs)

miRNAs are non-coding RNAs containing 18–25 nucleotides different from other RNAs [100,101]. mRNAs are involved in the regulation of oxidative stress-induced apoptosis [102]. They play essential roles in modulating gene expression and are involved in several cardiovascular disorders including cardiac hypertrophy [101,102,103]. Selected miRNA have regulatory effects on target gene expression. Thus, miR-34 family (miR-34a, -34b, and -34c) expression is up-regulated in different heart diseases. miR-153 regulates the survival of cardiomyocytes during oxidative stress through the modulation of apoptosis and autophagy directly mediated by targeting Mcl-1. This demonstrates the potential therapeutic role of miR-153 in the control of heart apoptosis [104]. Inhibition with antimiR-34a/antimiR-34 has also emerged as a promising therapeutic strategy with implications for cardiac drug development addressed to control heart hypertrophy and heart failure [103,105].

#### 3.4.9. G-Protein Signaling Pathway

G-proteins are part of is a multi-domain system that regulates signaling pathways with a pivotal role influencing pathologic cardiac hypertrophy and remodeling [106]. G-protein-mediated activation of MEK1/2-ERK1/2 signaling may be responsible for the pro-hypertrophic action of the regulator of G-protein signaling (RGS). RGS12 is a multi-domain member of the RGS family and plays a regulatory role in various signaling pathways, although the precise effect of RGS12 on cardiac hypertrophy remains largely unknown [107]. Therefore, regulation of G-protein pathways in cardiac hypertrophy through inhibition of RGS-10 [107] or RGHS6 [108] have been proposed as potential therapeutic targets to attenuate pressure overload-driven cardiac remodeling.

#### 3.4.10. Fibroblast Growth-Factor 21 (FGF21)

Experimental in vivo and in vitro studies in mice have shown that FGF-21 protects against cardiac hypertrophy. FGF21(−/−) mice exhibit enhanced induction of cardiac hypertrophy markers and pro-inflammatory pathways [33]. Furthermore, FGF21 is induced in failing human hearts [34] but human trials with FGF-21 have not yet been performed to actually demonstrate the protective role of FGF21 in heart diseases.

#### 3.4.11. Peroxisome Proliferator Activated Receptor Agonists (PPAR)

PPARs may protect against cardiac hypertrophy. PPARα agonists such as fibrates and PPARβ/δ agonists exert potent anti-hypertrophic and anti-inflammatory effects on the heart [109]. Pioglitazone, a peroxisome proliferator activated receptor (PPARγ) agonist protects against cardiac hypertrophy by inhibiting AKT/GSK3β and MAPK signaling pathways [110].

#### 3.4.12. Resveratrol

A natural plant product, also known as stilbenoid, selectively inhibits pathological cardiac signaling pathways and differentially regulates pathological cardiac hypertrophy [111]. Resveratrol inhibits NFAT-dependent transcription. The effects of low concentrations of resveratrol are AMPK-independent. According to these effects, resveratrol may be used in the complementary treatment of pathological cardiac hypertrophy.

#### 3.4.13. Alpha-Lipoic Acid (ALA)

ALA has been described as a therapeutic agent for a number of conditions related to cardiovascular disease [112]. ALA has a robust anti-hypertrophic and anti-remodeling effect that is mediated by inhibition of C/EBPβ activation [113]. Other authors have suggested that ALA partially attenuates cardiac hypertrophy via inhibition of PPAR2 and subsequent activation of Sirt1 and have proposed that it may have a potential cardio-protective role [112].

#### 3.4.14. BNIP-3

The Bcl-2/adenovirus E1B 19-kD interacting protein 3 (BNIP3) is a target in inflammation-mediated heart failure. It decreases heart apoptosis and may subsequently improve heart damage [81].

The strategies mentioned above are not yet available as regular treatments since they have not been tested in controlled clinical trials. Nonetheless, some of these approaches, such as myostatin, anti-miRNA and adrenergic receptor regulators, may be close to translation from the laboratory to the clinic. 

### 3.5. Control of Cardiac Fibrosis

Cardiac fibrosis is the excessive deposition of extracellular matrix (ECM), such as collagens and fibronectin, resulting in the accumulation of fibrous connective tissue [114,115,116]. Although fibrosis is essential in the biological repair process of damaged tissues, heart fibrosis is a pathological process that may induce stiffening, reduced oxygen diffusion, and is associated with arrhythmias, ventricular dysfunction and failure leading to pathological remodeling [117].

Replacement of lost cardiomyocytes by fibrotic material makes the environment less favorable, producing additional cardiomyocyte death. This also generates a vicious cycle of further decline of cardiac function [117]. This fibrotic process is implicated in almost all forms of heart disease [116].

The cardiac fibroblast is a relevant cell in the homeostasis of the ECM [118]. These cells actively synthesize connective tissue components and cause organ fibrosis [119]. Upon injury, stimulated, cardiac fibroblasts transform to a myofibroblast phenotype and develop a process of fibrocyte differentiation, epithelial to mesenchymal trans-differentiation, and endothelial to mesenchymal transition. Many different mediators and signaling pathways influence cardiac fibroblast function such as transforming growth factor beta (TGF-β), angiotensin II and platelet-derived growth factor (PDGF). Inhibition of the myofibroblast activation process may be useful to prevent cardiac fibrosis, including the use of these candidate proteins as treatment targets and the prospect of anti-fibrotic therapy either by systemic or localized delivery [120,121]. Breaking this fibrosis-cell death axis could further halt pathological cardiac regression and potentially reverse remodeling [117].

Ethanol is a direct highly active pro-fibrogenic molecule [25]. In addition to its direct effect, acetaldehyde adducts and lipid peroxidation products generated by ethanol metabolism also have pro-fibrogenic actions. Similar to that happens in the liver and pancreas, cardiac fibrosis is a relevant process in alcoholic cardiomyopathy, being present in both subendocardial and interstitial spaces usually in advanced stages of disease [28].

Despite the critical importance of fibrosis in cardiovascular disease, our limited understanding of the physiopathology of cardiac fibroblasts impedes the development of potential therapies that effectively target this cell type and its pathological contribution to disease progression [114,116]. Some mechanisms proposed to decrease fibrotic cardiac formation also share a similar influence on myocyte hypertrophy and cell loss, with the following being the most relevant:

#### 3.5.1. ROCK Inhibitors

In addition to the previously mentioned role on cardiac hypertrophy, ROCK inhibitors, such as fasudil contribute to controlling cardiac fibrosis and subsequent cardiac remodeling and heart failure [88,89]. However, there have been no human studies to corroborate this anti-fibrogenic role.

#### 3.5.2. miRNA

In relation to cardiac fibrosis, miR-21 and miR-29 are the agents most intensively studied. The miR-29 family is down-regulated in failing hearts and has been associated with several ECM-mediating encoding genes for fibrillin, elastin, and collagens under TGF-β regulation. miRNA have been proposed as biomarkers as well as targets for therapy [105].

#### 3.5.3. TGF-β Antagonists

TGF-β antagonism inhibits myocardial fibrosis by neutralizing anti-TGF-β antibodies or by proteoglycans and prevents the increase in gene expression of ECM proteins. This suggests a TGF-β mediated role in ECM protein production and fibrosis [121,122].

#### 3.5.4. Cytokines and Chemokines

In addition to TGF-β, several cytokines, chemokines, and growth factors are induced in the injured heart and may contribute to myofibroblast differentiation. Thus, endothelin-1, angiotensin II (Ang II), connective tissue growth factor (CCN2/CTGF), plateled-derived growth-factor (PDGF), serum response factor (SRF), transient receptor potential (TRP) channels, and mitogen-activated protein kinases (MAPKs) appear to act in a network that contributes to myofibroblast differentiation and persistence [118]. The chemokine Interferon-γ inducible Protein (IP)-10 exerts anti-fibrotic actions, inhibiting fibroblast migration [123]. Drugs targeting these proteins are currently under consideration as anti-fibrotic treatments [121].

#### 3.5.5. Relaxin

Relaxin is a cardioprotective agent that is up-regulated in heart disease. Relaxin may protect the heart via its anti-hypertrophic, anti-inflammatory, vasodilator and anti-fibrotic actions. This agent is an effective anti-fibrotic agent due to its specific ability to inhibit pro-fibrotic cytokine and/or growth factor-mediated fibroblast proliferation, differentiation and matrix production. It is able to down-regulate tissue inhibitor of metalloproteinase activity. Relaxin has been used in the treatment of non-systolic heart failure [124,125].

#### 3.5.6. Myostatin (Mstn).

Mstn induces interstitial fibrosis in the heart via TAK1 and p38. Therefore, Mstn inhibition may potentially protect against cardiac fibrosis [126].

#### 3.5.7. ω-3 Polyunsaturated Fatty Acids (ω-3 PUFAs)

Recent studies suggest that ω-3 polyunsaturated fatty acids (ω-3 PUFAs) inhibit cardiac fibrosis and attenuate diastolic dysfunction. This opens up possible new avenues for treatment of diastolic heart failure through the inhibition of cardiac fibrosis [127].

#### 3.5.8. Pioglitazone

Pioglitazone, a PPARγ activator has been reported to suppress cardiac fibrosis inhibiting the AKT/GSK3β and MAPK signaling pathways [110].

#### 3.5.9. Anthrocyanin

Purple rice anthocyanin extract may improve cardiac function by inhibiting cardiac hypertrophy and fibrosis [128].

### 3.6. Control of Oxidative-Energy Damage

**Figure 1:**
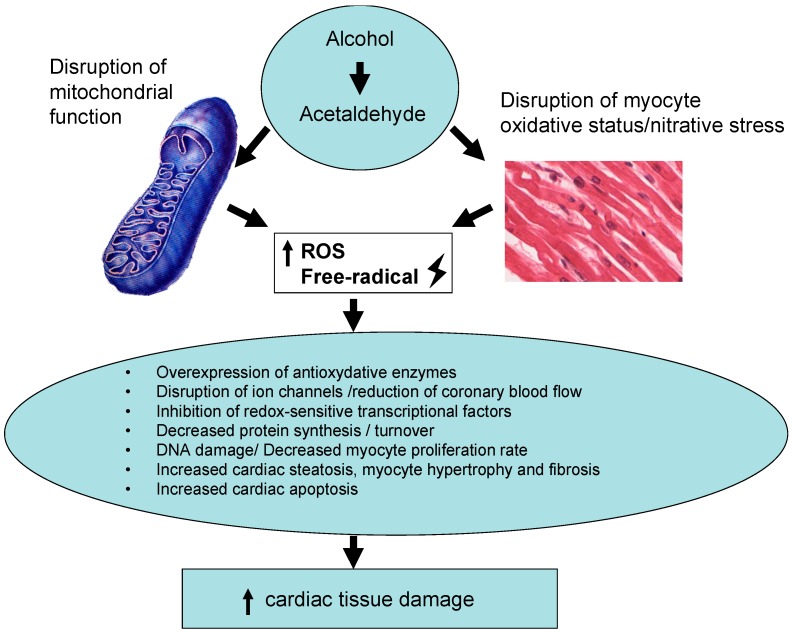
Role of reactive oxygen species (ROS) in alcohol-induced heart damage.

Oxidative pathways are one of the central mechanisms of the development of heart disease [79]. Chronic alcohol consumption disturbs heart mitochondrial function and oxidative status [20], resulting in an increase in ROS [21,129] (Figure 1). It may also increase the overexpression of anti-oxidative enzymes [130,131] and inhibit the redox-sensitive transcriptional factor (Nrf cascade), decreasing myocyte proliferation [132]. In a study with chronic alcoholic heart donors, the presence of dilated CMP was related to an increase in myocardial superoxide dismutase activity, a fact supposed to be a compensatory mechanism against alcohol-induced oxidative damage [133]. In the healthy heart, coronary blood flow is linked to the production of metabolites, which modulate smooth muscle tone in a redox-dependent manner. Some ion channels such as ATP-sensitive potassium (KATP) channels, voltage-gated potassium (Kv) channels, voltage-gated sodium (Nav) channels, and the L-Type ryanodine Ca^2+^ channel, among others, play a critical role in coupling myocardial blood flow to cardiac metabolism [18,134,135]. In fact, genetic polymorphisms or the absence of these channels disassociates metabolism from flow, resulting in tissue hypoxia, myocardial ischemia and cardiac pump dysfunction [134,135]. Thus, it would be interesting to determine whether genetic polymorphisms encoding for ion channels may be related to a major susceptibility to alcohol-induced myocardial ischemia and cardiac pump dysfunction [135,136]. Although antioxidant therapy seems indicated in alcohol-induced heart damage, clinical evidence of its utility is still low [79]. Some important approaches are discussed below.

#### 3.6.1. Novel Cardiomyokines

Myokines are a family of peptides produced, expressed, and released by myocytes [137,138]. They exert autocrine, paracrine or endocrine effects that may modulate the cardiac damage inflicted by different metabolic or toxic agents. Chemokine synthesis is stimulated by danger signals released from dying cells and damaged ECM, activating innate immune pathways [123]. Chemokines are involved in some contra-regulatory pathways, which emerge to modulate the intensity of cardiac oxidative damage [139].

Fibroblast growth factor-21 (FGF21) is a mediator of the mitochondrial pathways and has a relevant role in heart diseases [34]. It regulates ROS, superoxide dismutase-2 production and gene expression of the encoding proteins involved in anti-oxidative pathways. Considering this relevant pathophysiologic role, FGF21 has been proposed as a biomarker in mitochondrial damage in heart failure and heart damage [138]. The role of this emerging cardiomyokine in the pathogenesis of alcohol-induced heart damage may be relevant as a contra-regulatory pathway to modulate the intensity of heart damage [9]. In addition, FGF21 may be considered as a potential diagnostic marker of heart damage [139]. Therefore, the role of cardiomyokines in ACM oxidative damage is relevant, but needs further research.

#### 3.6.2. Leptin

Increased plasma leptin levels have been described in chronic alcohol consumption, producing heart and systemic oxidative damage [140]. Attempts to control this leptin-mediated oxidative damage in chronic alcoholics have been made with L-Carnitine and Ascorbic acid, with no clear results.

#### 3.6.3. Pioglitazone

In addition to anti-fibrotic activity, pioglitazone has been suggested to have antioxidant protective cardiac effects mediated by catalase against oxidative stress [141].

#### 3.6.4. Ghrelin

The endogenous ligand of growth hormone secretagogue receptor (GHS-R) ghrelin is a metabolic and energetic extra-hypothalamic regulator. It inhibits endoplasmic reticulum stress (ERS) and apoptosis and has cardio-protective properties [142]. Therefore, ghrelin could potentially be used to protect the heart against ERS-induced injury and apoptosis, and its potential therapeutic application has been suggested [143].

#### 3.6.5. Phenolic Compounds

The natural biphenolic compound Honokiol derived from the bark of magnolia trees has cardiac anti-oxidative properties with anti-hypertrophic effects due to activation of the deacetylase Sirt3 [144]. Similarly, *Leonurus. cardiaca* herb extract effectively attenuates the generation of free radicals in heart mitochondria and has been proposed asa useful remedy to protect cardiac muscles from oxidative damage [145]. Dietary supplementation with soy isoflavones increases eNOS activity and expression and activate the Nrf2-Keap1 signaling pathway. This leads to an up-regulation of detoxifying and antioxidant defensive genes with potential cardiac benefit. However, trials with isoflavone or phytoestrogens supply have largely reported only marginal health benefits [146].

Since the description of the so-called “French paradox” [147], in which low cardiovascular risk was related to red-wine consumption compared to other alcoholic beverages, many epidemiological studies [148,149] have suggested that polyphenols from red wine and other sources, mainly flavonoids, lignans and hydroxybenzoic acids, are able to decrease the global cardiovascular risk by 46% and of all-cause death by 37% [150]. However, clinical trials are needed to confirm this effect and establish specific recommendations [151]. 

## 4. Strategies to Improve Cell Regeneration and Repair

The adult heart is a terminal differentiated organ with very low regeneration power [35,152]. Regeneration of the injured myocardium is one of the most ambitious goals in modern cardiology [153]. A potential treatment strategy to improve injured cardiac tissues is enhancement of the endogenous regenerative capacity [35,153]. Recent reports have suggested that inflammation and different populations of cardiac macrophages might contribute to regenerative versus fibrotic responses [154].

### 4.1. Ki-67 and Myostatin

One marker of myocardial proliferation is Ki-67. The percentage of cardiac myocytes expressing Ki-57 in the nuclear area is an index of cardiac regeneration [155]. This Ki-67 percentage increases in all-cause cardiac damage as a compensatory response to damage. This is also the case of ACM in which the Ki-67 index is significantly increased in comparison to alcoholics without cardiac damage. However, high-doses of alcohol also inhibit myocardial proliferation, probably by *Mstn* up-regulation [9]. Thus, in chronic alcoholics the relative increase in Ki-67 percentage is 67% lower than in subjects with hypertension or other causes of CMP, evidencing a clear decrease in myocyte proliferation capacity in alcoholics. As a potential treatment target for ACM, Mstn inhibition could help to stimulate myocyte cell proliferation [156]. However, some limitations to this treatment still make it difficult to apply since Mstn inhibition produces glycolysis and increased glycogen storage and cardiac hypertrophy [157].

### 4.2. Telocytes

Cardiac telocytes support the stem cells for activation and commitment, and also help their migration toward injured myocardium. Telocyte reduction disturbs intercellular signaling and may participate in three-dimensional myocardium’s organization. Increasing telocyte function may help induce myocardial regeneration [158].

### 4.3. Stem Cell Therapy

The adult human heart has an extremely limited regenerative capacity, and there is minimal contribution from local progenitor cells [159]. In ACM, chronic alcohol consumption decreases the heart myocyte proliferation rate and contributes to decreasing this repair mechanism [35,36,155]. Cell therapy for heart repair has been performed using different cell types including skeletal myocytes, bone marrow mononuclear cells, mesenchymal stem cells, and cardiac-derived cells [160]. Bone marrow mononuclear cells for intracoronary cell therapy have been tested in different phase III trials after myocardial infarction but not in ACM. They showed non-homogeneous and diverse functional results [159,161]. A European BAMI multi-centric trial on the effect of intracoronary reinfusion of bone marrow-derived mononuclear cells is ongoing and may provide new data [162]. Mesenchymal stem cells (MSC), either autologous or allogeneic, have been established as one of the potential therapeutic agents in cardiac regeneration. They have been used for either revitalizing cardiac stem cells or revascularizing the arteries and veins of the heart mainly after acute myocardial infarction [163]. Most MSC studies have demonstrated that the cells die off within a week or two after transplantation, with little direct cardiac differentiation [159].

Recent advances using human stem cell-derived cardiomyocytes have been established as a next generation of cell type replacement therapy for moving forward. However, certain challenges must be overcome for this technique to be successful in the clinical practice [159].

Overall, this therapy is able to improve systolic and diastolic ventricle function and decrease inflammation and fibrosis. These beneficial effects are probably due to the indirect paracrine capacity of transplanted stem cells to facilitate endogenous cardiac repair processes, having demonstrated safety and modest efficacy in phase III clinical trials. At present, technical challenges still limit stem cell use in human studies [164,165], with these challenges including poor definition of the cell types used, the diversity in cell-handling procedures, and functional variability intrinsic to autologous-derived cells thus limiting factors of cell-based therapies [160].

### 4.4. Heart Transplantion

One final possibility to repair end-stage ACM is heart transplantation [166]. However, this is a limited strategy because chronic alcoholics with end-stage ACM (left-ventricle ejection fraction <15%) usually have liver cirrhosis, dementia or other systemic organ damage due to alcohol. In addition, a long period of alcohol abstinence should be guaranteed before heart transplantation. In a series of 94 chronic alcoholics with ACM, over a 59-month follow-up, 15% achieved heart transplantation [2].

## 5. Conclusions and Future Trends

In alcohol-mediated heart damage, the final result of dysfunction, structural damage and cardiac plasticity depends on a variety of factors, sometimes additive and occasionally counter-posed [12,14]. Therefore, new treatments in this field will necessarily be multidisciplinary and complementary or synergic in a personalized patient approach. Control of alcohol-induced systemic damage seems pivotal when approaching ACM [167,168]. Strategies combining a reduction of ethanol-dependent inflicted damage as well as increasing local and systemic protective mechanisms of myocyte repair will be necessary [9]. The development and application of new cardiomyokines (FGF21) [33,34,138] isoform-specific ROCK inhibitors [92] and MicroRNAs [105,169] will probably be combined to improve conventional therapeutic strategies such as control drinking [10,42], antioxidant supplementation [170], and anti-inflammatory [92,171], anti-fibrotic [120] and anti-apoptotic [172] systemic treatments as well as better use of stem-cell therapy [161] and heart transplantation [2,166] procedures.

## Figures and Tables

**Table 1:** Mechanisms of alcohol-induced heart damage.

Mechanisms	Effectors
Interference with cell signaling and calcium transients [16,17]	MAPK, TGF-β, PKC, PPARγ, MMPs, NF-κβ, PAI-1
Decrease in excitation-contraction coupling mechanisms [17,18,19]	intracellular [Ca]^2+^ transients, L-type Ca^2+^ channel
Induction of oxidative damage [20,21]	ROS, SOD, acetaldehyde
Pro-inflammatory effect [22]	IL-2, TNF-α, NF-κβ
Induction of apoptosis [23,24]	FAS, TNF-α, TGF-β, Bax-Bcl-2, caspases 3,6
Induction of fibrosis [25]	TLR-4, TGF-β
Protein-adduct formation [26]	protein-ethanol-adducts
	malondialdehyde-DNA adducts
Disruption in protein synthesis [27]	decrease in ribosomal protein synthesis, actin, myosin, troponin, titin
Increased glycogen deposition [28,29]	glycogen synthase kinase-3β, PARP
Renin-angiotensin-aldosterone activation [30]	renin, angiotensin, aldosterone, p38 MAPK/Smad
Interference in hormone-growth factors [31,32]	myostatin, ghrelin, leptin, IGF-1
Interference in regulatory cardiomyokines [33,34]	FGF21
Decrease in myocyte regeneration [35]	myostatin, IGF-1
Impairment of extracellular matrix turnover [16]	cytoskeletal structure, connexin channel, desmosome contacts
Imbalance between cardiac lesions/repair mechanisms [9]	cell apoptosis and necrosis increased myocardial fibrosis decreased myocyte regeneration

**Table 2:** The effects of high-dose alcohol on short and long-term cardiovascular damage.

Short-Term Effects on the Heart	Long-Term Effects on the Heart		Long-Term Effects on the Vascular System	
Dysfunction of cardiac contractility	Ventricular dysfunction	Diastolic dysfunction	Increased systemic atherosclerosis	
		Systolic dysfunction		
Acute arrhythmias supraventricular ventricular (holiday heart syndrome)	Atrial dysfunction		Arterial hypertension	
Arterial hypertension	Chronic arrhythmias		Peripheral artery disease	
Transitory ischemic cerebral attack	Alcoholic cardiomyopathy	Subclinical cardiomyopathy	Changes in lipid profile	Increase in LDL cholesterol
		Clinical congestive heart failure		Increase in triglycerides
		Low-output dilated cardiomyopathy		
Sudden death	Coronary heart disease	Angina	Increased risk of diabetes	
		Myocardial infarction		
	Increased cardiovascular mortality		Interference with other cardiotoxic drugs (tobacco, cocaine)	
